# Screening Candidate Genes for Body Size Traits in Dongfeng Sika Deer Bucks Based on Genome-Wide Association Analysis

**DOI:** 10.3390/biology15030227

**Published:** 2026-01-26

**Authors:** Yan Zhang, Xinyuan Zhang, Lieping Zhao, Zhen Zhang, Yao Zhao, Wenxi Qian, Guanghui Gai, Huixin Bai, Peize Du, Huansheng Han

**Affiliations:** 1 College of Animal Science and Veterinary Medicine, Heilongjiang Bayi Agricultural University, Daqing 163319, China; zhangyan990625@163.com (Y.Z.); zhangxinyuan011015@163.com (X.Z.); duyiqi77@163.com (P.D.); 2 Heilongjiang Academy of Land Reclamation Sciences, Harbin 150038, China; zhaolieping66@163.com (L.Z.); dongnongzz@126.com (Z.Z.); 3 School of Life Sciences and Technology, Harbin Normal University, Harbin 150025, China; yaozhao829@163.com; 4 College of Animal Science and Technology, Tarim University, Alar 843300, China; qianwenxizj@163.com; 5 Inner Mongolia Genhe Forest Industry Co., Ltd., Genhe 022350, China; nmsgggh@163.com; 6 Daqing Municipal Agricultural and Rural Social Service Center, Daqing 163311, China; 39357009@163.com

**Keywords:** Dongfeng sika deer and buck, genome-wide association analysis, body size traits, candidate gene

## Abstract

This study aimed to screen candidate genes related to body size traits in Dongfeng sika deer bucks by genome-wide association analysis. A total of 266 adult Dongfeng sika deer underwent 20× whole-genome resequencing. Genome-wide association analysis (GWAS) was performed using a mixed linear model (MLM), and gene annotation was conducted on SNP loci. GO and KEGG enrichment analyses were subsequently conducted to identify biological functions and pathways associated with body size regulation. The GWAS results screened 774 SNP sites significantly associated with body size. Functional enrichment analysis further identified that four genes—*CDH4*, *TSHR*, *SLC23A2*, and *RIMS1*—were potentially associated with body size traits. This study provides a reference basis for subsequent functional exploration of candidate genes.

## Simple Summary

The aim of this study was to screen candidate genes associated with body size traits in Dongfeng sika deer stags by genome-wide association analysis. Sika deer is one of the most important deer species in China, and the body size trait directly affects its meat production performance and reproductive efficiency. However, research on body size traits of sika deer is still limited. To address this issue, we sequenced the whole genome of 266 adult Dongfeng sika deer stags and screened for genetic markers associated with nine body size traits by genome-wide association analysis. A total of 774 genetic loci with significant association with body size traits were detected, and four candidate genes that could positively regulate the development of body size were identified after further analysis. The results of this study provide new genetic markers and a theoretical basis for investigating the genetic mechanism of body size and meat production traits in sika deer, and provide an important reference for selecting and breeding better sika deer populations. Ultimately, this could help improve the economic benefits of deer farming through scientific breeding strategies.

## 1. Background

The Sika deer (*Cervus nippon*) is a member of the class Mammalia, order Artiodactyla, family Cervidae, and genus Cervus. It is among the most abundant, diverse deer species in China, and represents one of the earliest domesticated deer in the country. In recognition of its genetic and economic importance, it was listed in the National Catalogue of Livestock and Poultry Genetic Resources in 2020 [1]. Over the years, extensive breeding programs have been carried out, including Shuangyang sika deer, Xifeng sika deer, Aodong sika deer, Siping sika deer, Xingkaihu sika deer, Dongfeng sika deer, Dongda sika deer, and Changbaishan sika deer, which have been cultivated [2]. Among these sika deer, the Dongfeng sika deer is characterized by its purebred status, stable genetic performance, good disease resistance, high tolerance, and excellent production performance [3]. In recent years, China has mainly focused on breeding antlered sika deer, while less research has been conducted on breeding meat sika deer. However, with the increasing use of livestock and the expansion of the venison market, interest in improving sika deer meat yield has grown substantially among breeders. As a major economic trait in the production of sika deer, genetic selection for body size is the main goal of meat sika deer breeding, and at the same time, body size has a direct relationship with productivity and fitness, which has a decisive impact on the efficiency of meat sika deer production.

The Genome-Wide Association Study (GWAS) approach is an effective tool for identifying genetic markers linked to economically important traits in plants and animals. By detecting marker loci associated with phenotypic variation, GWAS facilitates marker-assisted selection. This, in turn, enables the improvement of livestock breeds, enhancement of production performance, and optimization of economic efficiency. Currently, GWAS has become one of the most important tools for research in livestock population genetics and gene mining of economically important traits [4]. The Mixed Linear Model (MLM) in the GEMMA software (v0.98.5) can correct for both population structure and individual relatedness and reduce computational time. For instance, Li [5] identified 16 single-nucleotide polymorphism (SNP) loci associated with four growth traits, including buck body weight, buck body height, doe body slant length, and doe hip height, and annotated 15 candidate genes influencing growth and body development. Excellent body size traits can improve livestock production performance. Excellent body size traits can improve the production performance of livestock and increase the economic benefits of farmers, and therefore have always been an important parameter in breeding work [6]. Despite its importance, research on body size traits in sika deer remains limited. To date, there have been few comprehensive studies aimed at screening genes associated with body size in this species. At present, research on sika deer mainly focuses on differentiation among sika deer, equids, and their hybrids [7,8], reproductive physiology [1], transcriptomic sequencing of genes involved in antler growth [9,10,11], and selection signals for reproductive traits [12]. However, the genetic mechanisms underlying body size variation in the Dongfeng sika deer remain largely unexplored, and systematic investigations at both quantitative and molecular genetic levels are still lacking. This study aims to identify SNPs and associated candidate genes significantly linked to body size traits in male Dongfeng sika deer through genome-wide association analysis. The aim of this study was to screen the SNP loci and candidate genes that were significantly associated with the body size trait of Dongfeng sika deer bucks by the MLM based on genome-wide association analysis, so as to provide a reference for the subsequent investigation of the function of the candidate genes.

## 2. Materials and Methods

### 2.1. Test Animals and Sample Collection

In 2024, we selected 266 five-year-old Dongfeng sika deer bucks from the Dongfeng County Dongfeng Sika Deer Seed Source Co. deer farm (Dongfeng, China). All selected animals were in good body condition and under consistent feeding management. A single surveyor used measuring rods and tape measures to take phenotypic measurements, including body height (HT), body length (BL), chest circumference (WS), chest depth (CC), head length (HL), frontal width (FW), pipe circumference (PC), tail length (WC), and antler shank distance (JB). All measurements were conducted and recorded in accordance with the “Technical Specification for Determination of Productive Performance of Antlered Deer” (NY/T1179-2006). Data points lying outside the range of the mean ± 3 standard deviations were treated as outliers and excluded prior to statistical analysis. Jugular blood samples were collected from each animal and stored at −80 °C for subsequent molecular analyses.

### 2.2. DNA Extraction and Library Sequencing

Genomic DNA was extracted using the Sevin Biological Reagent Kit (model SD011, Sevenbio Innovation (Beijing) Biotechnology Co., Ltd., Beijing, China). The purity and integrity of DNA samples were analyzed by agarose gel electrophoresis, and concentration was determined spectrophotometrically by measuring the OD260/OD280 ratio, which ranged from 1.7 to 1.9. Qualified DNA samples were sent to the Smartgenomics Technology Institute (Tianjin, China) for sequencing. DNA was randomly fragmented to approximately 350 bp using a Covaris ultrasonicator (Covaris, LLC, Woburn, MA, USA). The resulting fragments underwent end-repair, A-tailing, adapter ligation, purification, and PCR amplification for library construction. After quality assessment, sequencing was performed according to the library’s effective concentration and data output requirements.

### 2.3. Genotype Quality Control and Principal Component Analysis

In the data preprocessing stage, the raw reads generated by sequencing were quality controlled using fastp (v0.20.0), with filtering criteria including: trimming of splice sequences, elimination of read pairs with a number of ambiguous bases (N) greater than 5 in single-ended reads, and elimination of read pairs with a percentage of low-quality bases (Q ≤ 15) greater than 40%. The post-QC Clean reads were compared to the reference genomes of homologous species using Senticon software (v202112) with default parameters, and sorted and de-weighted using Samtools. Based on the processed comparison results, Sentieon was used to perform initial screening for single-nucleotide polymorphisms (SNPs) with the following criteria: sequencing depth support of not less than 4, comparison quality value of not less than 40, and genotype quality value of not less than 5.

Population-level genotypic data were rigorously filtered at multiple levels to obtain high-quality datasets for association analysis: genotypes with genotype quality values (GQ) greater than 20 and depth of coverage greater than 5 were retained; filtration was performed using PLINK (v1.90) to retain only bi-allelic loci. Loci with a missing-genotype rate exceeding 20% were removed, ensuring that at least 80% of the population was genotyped at each SNP. Variants with a minor allele frequency (MAF) greater than 0.05, and Hardy–Weinberg equilibrium (HWE) *p*-values greater than 1 × 10^−6^. Principal component analysis was performed using GCTA (1.94.1) software, and the first 2 principal components were used as covariates in subsequent association analyses to eliminate the effect of population structure on the analysis. After the above process, a total of 27,127,458 high-quality SNPs were considered for further analysis.

### 2.4. Genome-Wide Association Analysis

This study conducted genome-wide association analysis between body size traits and SNP loci of Dongfeng sika deer bucks, using the mixed linear model (MLM) in GEMMA v0.98.5. To account for potential confounding effects, population genetic structure was included as fixed effects in the model, while the kinship matrix among individuals served as random effects. The significance threshold was set at −log_10_(P) = 7.5. The mixed linear model used was:

y = *Xα* + *Zβ* + *Wμ* + e

y is the phenotypic trait, *X* is the association matrix of fixed effect vectors, and *α* is the fixed effect vector; *Z* is the SNP genotype indicator variable, and *β* is the effect vector of SNPs; *W* is the design matrix of polygenic effect vectors, and *μ* is the microeffective polygenic effect vector obeying the normal distribution *μ*~N(0, *K*σ_g_^2^), where *K* is the genomic kinship matrix and σ_g_^2^ is the additive genetic variance; e is the random residuals, obeying e~N(0, Iδ_e_^2^), where I is the unit matrix and δ_e_^2^ is the residual variance.

### 2.5. Multiple Testing Hypotheses

Significant SNP loci were identified using the Bonferroni correction method. Linkage disequilibrium pruning was performed on SNPs via PLINK to determine the number of independent markers (N). The threshold of significance at the genomic level was defined as 0.05/N, while the threshold of significance at the latent level was set at 1/N, with N being the total number of SNPs. In this study, the threshold was set at 7.5 (P = 1/N). The genome-wide significance threshold was set at 7.5 (P = 1/N), and significant loci associated with body size traits were selected.

### 2.6. Enrichment Analysis of Annotated Genes

The window was set to 100 kb, the step size was 10 kb, and the analysis was performed with a sliding 50% overlap region. The Fst values were sorted from largest to smallest, and the top 5% regions with a high degree of differentiation were used as strong candidate regions. Haplotype analysis was performed on the remaining variant loci, and the haplotypes were defined according to the linkage disequilibrium (LD) pattern. After multiple comparisons of phenotypic values between different haplotypes, haplotypes whose genes exhibited significant variation were retained as the final candidate gene set. Candidate genes were annotated within a 0.1 Mb window upstream and downstream of significant SNP loci using ANNOVAR software (v20191024). Functional annotation and pathway enrichment analyses of these candidate genes were performed using the Metascape platform and the KOBAS online tool.

### 2.7. Data Statistics

Body size trait data were organized in Excel, the coefficient of variation (standard deviation/mean) was calculated, and the data were statistically analyzed using SPSS 26.0.

## 3. Results

### 3.1. Measurement Statistics of Body Size Traits

**Figure 1:**
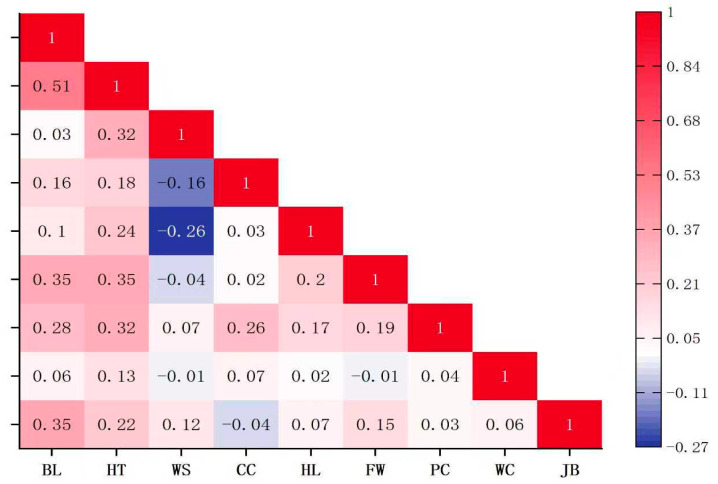
Matrix of body size traits of Dongfeng sika deer bucks: The color gradient corresponds to the strength and direction of the Pearson correlation coefficient (ranging from −0.27 to 1.0; red indicates a positive correlation, blue a negative correlation).

Statistical analysis of body size traits in 266 Dongfeng sika deer bucks was conducted, and the results are shown in Table 1. Correlation analysis revealed the relationship between traits related to body size of 266 Dongfeng sika deer bucks, and the results are shown in Figure 1. The coefficients of variation for the measured traits ranged from 4.71% to 28.40%, with chest circumference exhibiting the highest coefficient of variation, indicating a wide phenotypic dispersion; head length also showed substantial variability and suggests greater potential for genetic improvement.

### 3.2. Results of Principal Component Analysis

**Figure 2:**
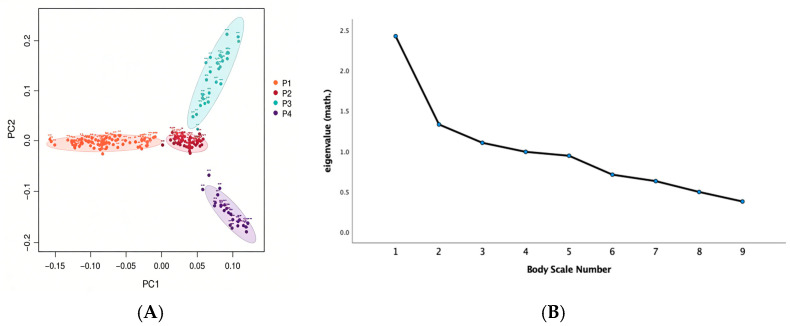
Principal component analysis: (**A**) principal component analysis. (**B**) Scree plot showing the eigenvalues (*y*-axis) of the first nine principal components (*x*-axis): number 1 to 9: body height (HT); body length (BL); chest circumference (WS); chest depth (CC); head length (HL); frontal width (FW); pipe circumference (PC); tail length; and antler shank distance (JB).

The results of principal component analysis (PCA) are presented in Figure 2. Figure 2A indicates some stratification within the population. To determine the optimal number of principal components (PCs) to retain, we employed a combined approach using a scree plot and the Kaiser criterion. The scree plot (Figure 2B) exhibited a typical decreasing trend, with the first three PCs having eigenvalues of 2.42, 1.85, and 1.32. A clear inflection point was observed after the third PC. Cumulatively, these first three PCs explained 54.03% of the total variance, sufficiently capturing the major structure of the data. Therefore, the first three principal components were retained in this study for subsequent group structure analysis and GWAS covariate correction to eliminate the influence of group stratification effects on the results of correlation analysis and to improve the accuracy of the results.

### 3.3. GWAS Results of Body Size Traits of Dongfeng Sika Deer Bucks

**Figure 3:**
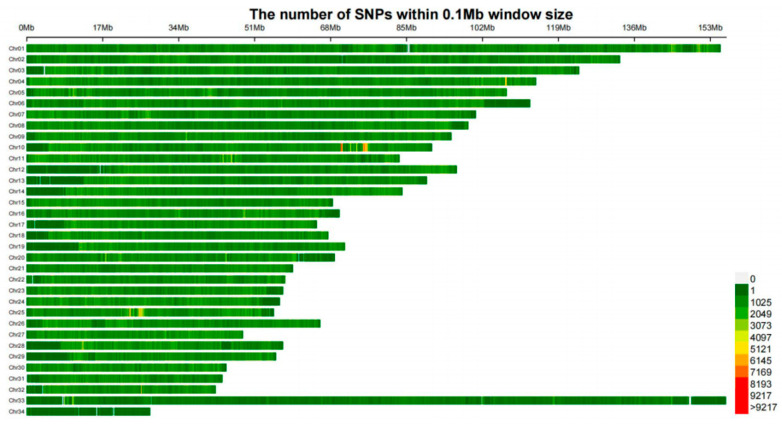
Heatmap of SNP distribution: The different colors in the right measurement represent the number of SNPs within the 0.1 Mb window size.

**Figure 4:**
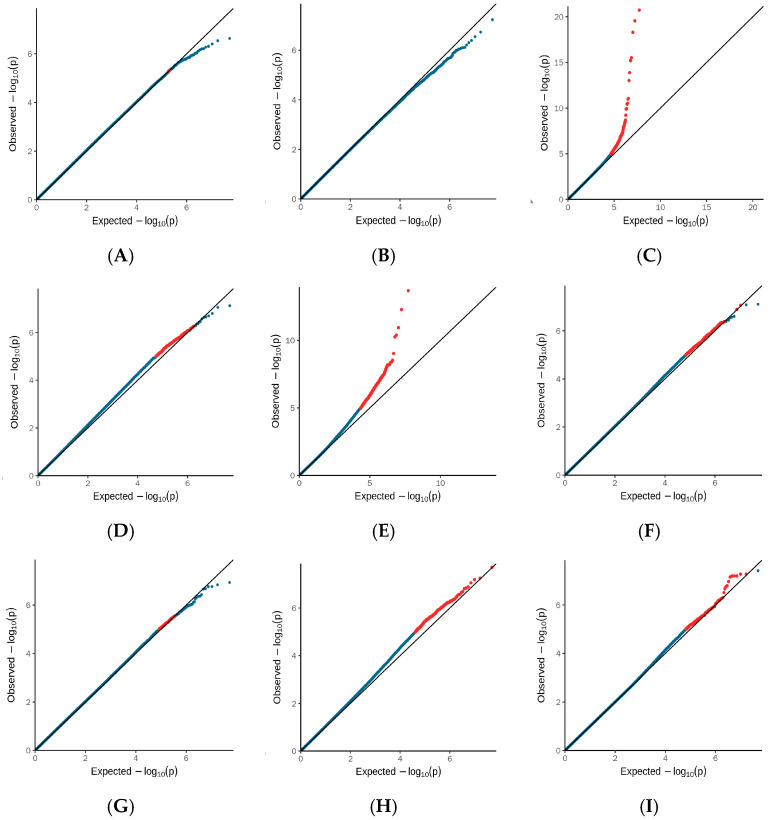
Dongfeng Sika Deer QQ Diagram: (**A**) body height (HT). (**B**) body length (BL). (**C**) chest circumference (WS). (**D**) chest depth (CC). (**E**) head length (HL). (**F**) frontal width (FW). (**G**) pipe circumference (PC). (**H**) tail length (WC). And (**I**) antler shank distance (JB). Blue dots in the figure represent SNP loci that do not reach the suggestive significance level, while red dots represent SNP loci that reach the suggestive significance level or genome-wide significance level.

**Figure 5:**
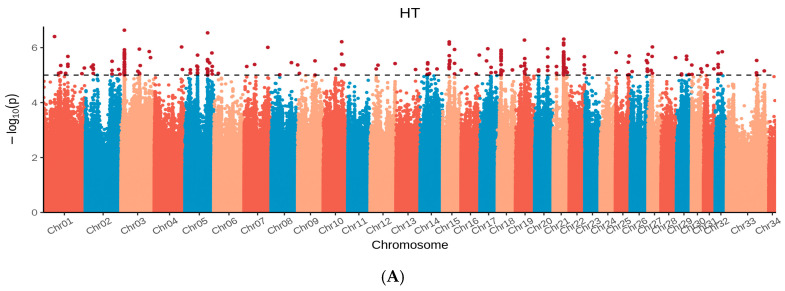

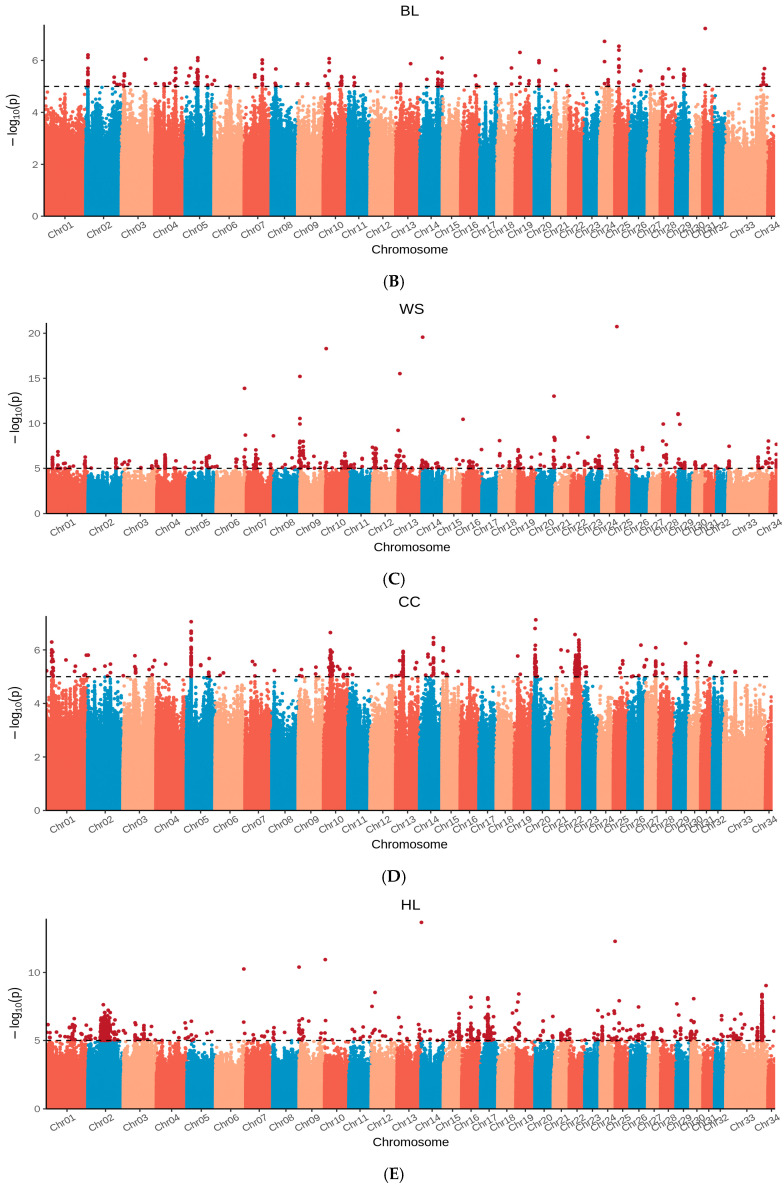

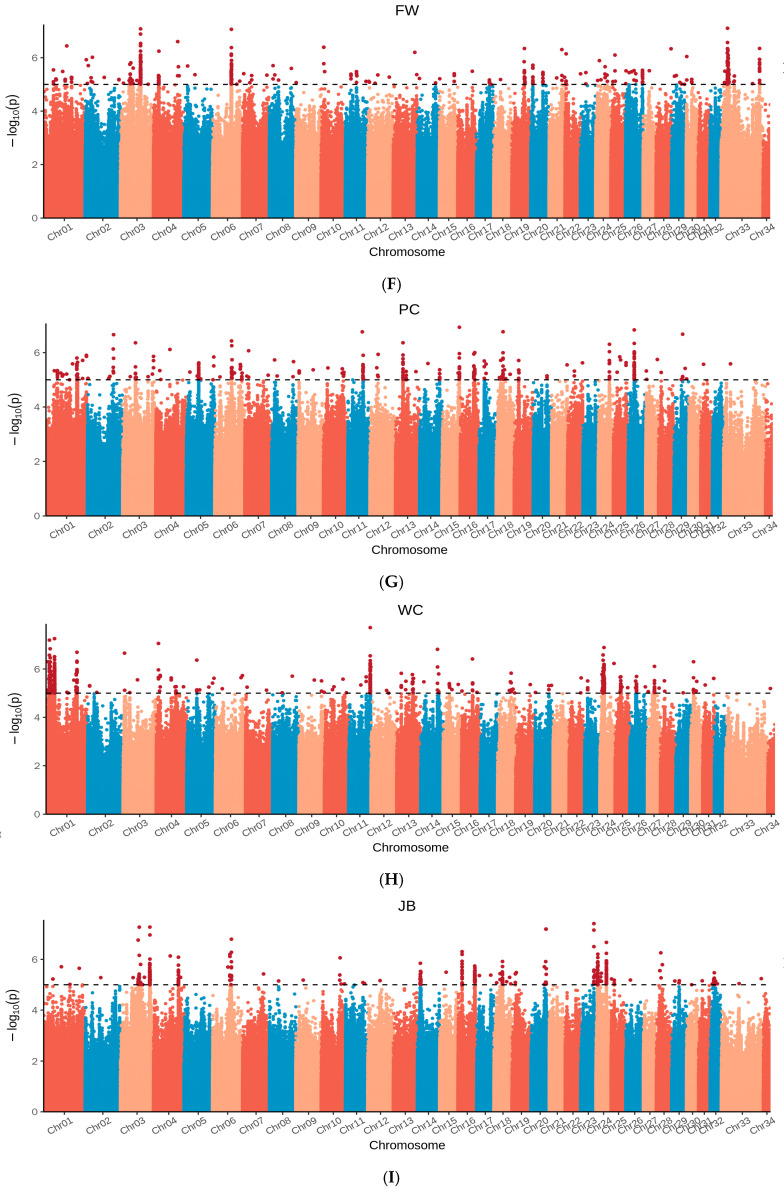
Manhattan plot of genome-wide association study: (**A**) body height (HT). (**B**) body length (BL). (**C**) chest circumference (WS). (**D**) chest depth (CC). (**E**) head length (HL). (**F**) frontal width (FW). (**G**) pipe circumference (PC). (**H**) tail length (WC). And (**I**) antler shank distance (JB), The black dashed line represents the genome-wide significance threshold after Bonferroni correction.

The density map of SNP and principal component analysis distribution after quality control is presented in Figure 3. Genome-wide association analysis for 9 body size traits using the GEMMA model is shown in Figure 4 and Figure 5, and selected SNP loci associated with these traits are summarized in Table 2. Detailed information on the SNPs associated with body length (BL) and the loci of SNPs associated with other body size traits are provided in Supplementary Tables S1 and S2, respectively. After quality control of the genotyping data from the 266 Dongfeng sika deer bucks using PLINK v1.90, a total of 27,127,458 high-quality SNPs were retained for GWAS. A total of 774 significant SNP loci (−log_10_P = 7.5) were screened in the GWAS analysis of Dongfeng sika deer bucks. Chest circumference and head length had the highest number of associated loci, 664 and 94, respectively, and the number of significant loci for the rest of the traits ranged from 0–7, including 6 for body slant length, 1 for frontal breadth, 2 for tube circumference, and 7 for antler shank spacing, while no significant loci were found for body height, breast depth and tail length. In the gene annotation, some loci were not found within 100 kb upstream and downstream of mCerEla1.1 (annotated version Release 100) of the horse deer (Cervus elaphus) genome, and were labeled as none, whose functions need to be further investigated.

### 3.4. Functional Annotation of Candidate Genes

**Figure 6:**
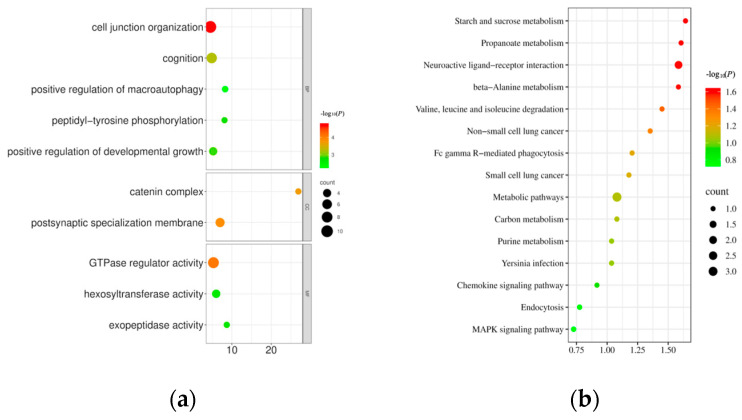
Bubble plot of candidate genes enrichment analysis: (**a**) GO gene functional annotation. (**b**) KEGG pathway enrichment analysis.

Gene Ontology (GO) and Kyoto Encyclopedia of Genes and Genomes (KEGG) enrichment analyses were performed on the candidate genes, and the results are presented in Figure 6, Table 3 and Table 4. The GO enrichment analysis showed that the candidate genes were enriched in three major categories: biological process (BP), cellular component (CC), and molecular function (MF). Among the significantly enriched GO terms, 10 were strongly associated with growth and developmental regulation. In particular, the *CDH4*, *TSHR*, *SLC23A2*, and *RIMS1* genes are putative candidate genes derived from these enrichment results; these genes were annotated to be associated with the positive regulation of developmental and growth processes. The KEGG pathway enrichment analysis identified 15 signaling pathways, none of which reached the threshold for statistical significance (corrected *p* < 0.05). However, the MAPK signaling pathway was found to be enriched in the *MAP3K20* gene, which has been reported to be involved in the regulation of growth and development [13,14,15,16,17,18,19], and this finding suggests that this pathway may be a potential direction that deserves in-depth validation in *Merganseria* spp. However, its specific role in this study is still speculative and needs to be confirmed by future expression analysis and functional experiments.

## 4. Discussion

The identification of candidate genes associated with economically important traits at the genome-wide level has become an important tool in animal breeding. Among available methods, selection signal detection provides an effective means to rapidly and accurately identify genomic regions under selection pressure and localize functional gene variants that may influence specific traits [20]. This approach ensures that genetic variations among populations and screen candidate genes for superior traits can be screened and identified. This will provide a lot of reliable basic data for the subsequent validation of candidate genes related to body size traits in sika deer.

GWAS plays an important role in the study of complex traits in plants and animals, especially in livestock and poultry breeding. Analyzing genetic diversity in livestock and poultry helps elucidate gene-trait relationships and underlying genetic architecture, enabling the effective identification of trait-associated SNPs and the localization of corresponding genomic regions [21,22]. Body size in livestock and poultry serves as a direct indicator of overall growth and development and correlates strongly with production performance, disease resistance, and the animals’ ability to adapt to environmental conditions [23]. Li [5] identified 16 SNP loci associated with four body size traits—buck body weight, buck body height, doe body slant length, and doe hip height—and annotated 15 candidate genes. In this preliminary study, a total of 774 SNP loci were identified as significantly associated with body size traits across nine phenotypic measurements in Dongfeng sika deer bucks. GO enrichment analysis revealed four candidate genes involved in the positive regulation of developmental and growth processes. Although no pathways reached the statistical significance threshold in the KEGG enrichment analysis, the MAPK signaling pathway, in which *MAP3K20* was enriched, was notably associated with growth and development. This research differs from previous studies in that it enriches the genetic marker library and functional gene resources for a specific group of Dongfeng sika deer bucks, advancing from broad-spectrum exploration to precise, deep excavation, and Data support for further subsequent validation. Based on the identified genes (*CDH4*, *TSHR*, *SLC23A2*, *RIMS1*) and the MAPK pathway associated with *MAP3K20*, the results provide insight into the molecular mechanisms underlying body size regulation in sika deer. These findings align with known developmental pathways in other Artiodactyla species and can be echoed in the mechanism of body size regulation in Artiodactyls. Collectively, this study provides a basis for the future integration of genetic resources and the development of a breeding technology chain.

The *CDH4* (Cadherin 4, calcineurin gene) was first isolated from chicken retina and identified as a retinal cadherin gene. As a member of the calmodulin family, *CDH4*, like other family members, functions in different tissue developmental and physiological processes by mediating calcium-dependent adhesion to cell surfaces. The functions of the cadherin family are evolutionarily conserved across species. In the nervous system, *CDH2* and *CDH4* expression accompany the development of neuronal synapses in the chicken brain [24]. In epithelial and reproductive tissues, the high concentration of calreticulin in bovine oviduct epithelial cells and gametes supports the conserved role of the calreticulin family in regulating physiological functions through cell–cell interactions [25]. In addition, *CDH4* has been reported to regulate epithelial cell proliferation [26] and to play an important role in the embryonic and postnatal development of multiple tissues and organs [27]. Functionally, *CDH4* is involved in cell adhesion and proliferation—processes critical for the development of body, skeletal, and other size-related tissues. Consistently, in our study, phenotypic variation among Dongfeng sika deer bucks, particularly in chest circumference and body slant length, likely stems from developmental differences in pectoral muscles and trunk bones. This alignment strongly supports the relevance of CDH4’s biological role to the observed traits. Furthermore, *CDH4* was significantly enriched in the “positive regulation of growth” GO term in this study. Taken together, these observations suggest that *CDH4* is a key candidate gene influencing body size traits in sika deer and may serve as a theoretical reference for understanding the molecular mechanisms governing growth and development in this species.

*TSHR* (thyrotropin receptor) is a member of the G protein-coupled receptor family and belongs to the leucine-rich repeat subfamily (LGR family). It serves not only as a characteristic transmembrane receptor on the surface of thyroid follicular cells but also functions as a regulatory marker in adult stem cells, where it contributes to tissue growth and differentiation [28]. Its core function is to initiate the growth, proliferation, and differentiation of thyroid cells by binding TSH. This interaction activates phospholipase C and protein kinase A signaling cascades via the second messenger cAMP, establishing a regulatory axis of “receptor–signaling pathway–cellular response” that directly governs thyroid development and thyroid hormone synthesis [29,30]. The thyroid gland is a key regulator of growth and metabolism. It indirectly affects skeletal development and overall body size by controlling growth rate, neurodevelopment, and whole-body metabolism. These effects are closely linked to variations in body height and body length among Dongfeng sika deer bucks. Recent studies have shown that *TSHR* expression is not limited to thyroid cells but is also widely expressed in lymphocytes, adipocytes, myofibroblasts, and gonads [31,32] and that *TSHR* deficiency leads to severe growth retardation and intestinal dysplasia in mice [33]. This cross-species functional validation reinforces the extensive and indispensable functions of *TSHR* in regulating growth and tissue differentiation. It provides strong support for the hypothesis that *TSHR* influences body size traits in sika deer through a dual mechanism involving thyroid hormone regulation and synergistic expression across multiple tissues. Consequently, this study’s findings validate *TSHR* as a critical candidate gene for body size traits in sika deer and lay the foundation for future functional validation and molecular breeding strategies.

*SLC23A2* (Solute Carrier Family 23 Member 2) encodes the sodium-dependent vitamin C transporter 2 (SVCT2), which plays a crucial role in regulating the transport and utilization of vitamin C (ascorbic acid) within the body. SVCT2 is essential for maintaining vitamin C homeostasis in tissue cells and contributes to the modulation of several physiological and pathological processes [34]. Experimental deletion of *SLC23A2* in mice leads to a marked reduction in SVCT2 expression in the brain, accompanied by downregulation of multiple associated proteins and brain-derived neurotrophic factors, and upregulation of Ionized Calcium Binding Adaptor Molecule 1 (Ibal). These alterations result in elevated oxidative stress and inflammation-related indices, including changes in glutathione levels and reduced vitamin C concentration in brain tissues. Such evidence indicates that *SLC23A2* is indispensable for fetal brain development and provides a robust model for investigating the physiological functions of vitamin C in neural development [35]. These findings not only highlight the indispensable function of *SLC23A2* in fetal brain maturation but also elucidate its broader regulatory mechanism operating through the “vitamin C transport–oxidative stress/inflammation modulation–tissue development” axis. Moreover, *SLC23A2*-mediated vitamin C transport appears to influence antioxidant equilibrium and myofibrillar protein synthesis within myoblasts. Therefore, the enrichment of *SLC23A2* in the present study suggests its potential association with body size traits and can be further verified at the pathway and molecular levels, thereby providing a direction for exploring the specific mechanisms underlying the association between genes and traits.

*RIMS1* (Regulating Synaptic Membrane Exocytosis 1) encodes a protein belonging to the RAS gene superfamily, primarily functioning in the regulation of synaptic vesicle exocytosis. This process is crucial for neurotransmitter release and underpins several neurophysiological activities, including modulation of voltage-gated calcium channels and regulation of long-term synaptic plasticity, thereby providing a multidimensional molecular basis for trait association. In terms of tissue-specific functions, the *RIMS1* protein plays a vital role in retinal synapses by precisely modulating neurotransmitter release to ensure accurate transmission of light signals from photoreceptor cells to downstream neurons [36], underscoring the necessity of this gene for the maintenance of physiological functions in specific tissues. Studies have shown that while the brain structure of *RIMS1*-knockout mice appears normal under microscopy, these animals exhibit severe growth retardation and premature death [37]. Short et al. [38] also demonstrated that de novo mutations in *RIMS1* were directly associated with neurodevelopmental disorders, revealing that they cause disease through, among other things, abnormalities in synaptic transmission and are associated with developmental delay. These findings collectively highlight the central role of *RIMS1* in neuroregulation, growth, and overall development. The link between *RIMS1* and animal body size may stem from its indirect effect on neuromuscular balance. This could involve regulating nutrient metabolism and muscle growth rate. This study’s finding that *RIMS1* is associated with body size traits in Dongfeng sika deer bucks supports this idea. Therefore, these results provide compelling evidence for *RIMS1* as a potential candidate gene linked to growth-related body measurements.

MAPK (mitogen-activated protein kinase) is a serine/threonine protein kinase widely distributed in eukaryotic cells that can be activated by extracellular signals and regulates growth and body measurements through a tertiary signaling cascade of “MKKK–MKK–MAPK”. The classical MAPK pathways include JNK, p38 MAPK, and ERK, corresponding to the three core subfamilies of ERK, JNK/SAPK, and p38 MAPK in mammals, and are involved in inflammation, oxidative regulation, muscle development, and various disease processes [13,14,15,16,17,18]. *MAP3K20* (also known as ZAK/MLTK) is a key upstream regulator of the MAPK signaling pathway [39,40]. It activates the JNK and p38 MAPK cascades by phosphorylating the downstream MEK and functions in a tissue-specific manner. In skeletal muscle, *MAP3K20* promotes protein synthesis and myofibrillar growth through the PTEN/AKT and mTORC1 axes. Experimental evidence shows that muscle-specific knockdown of *MAP3K20* leads to a marked decrease in skeletal muscle mass and strength in mice, whereas its overexpression protects muscle tissue from atrophy. Beyond its role in muscle biology, *MAP3K20* is also implicated in maintaining intestinal homeostasis and regulating lipid metabolism [19]. In this study, enrichment analysis linked *MAP3K20* and other MAPK pathway genes to body size traits in sika deer. Although the association identified by this study through enrichment analysis between *MAP3K20*, MAPK pathway signaling network-related genes, and the body size traits of sika deer did not reach statistical significance, prior evidence linking *MAP3K20* and the MAPK pathway to growth and developmental processes suggests a biologically relevant relationship. Given the well-documented role of this pathway and *MAP3K20* in animal growth and development [13,14,15,16,17,18,19], the presence of these elements in the enriched list not only highlights potential directions for further research but also implies that this pathway and its related genes may exert a functional role in the regulation of body size in sika deer. The lack of statistical significance may stem from the polygenic and micro-effect nature of body size traits, where the cumulative influence of numerous genes may not be sufficient to reach the genome-wide threshold. Moreover, *MAP3K20*, while a key upstream activator within the MAPK cascade, may indirectly influence body size through “signaling pathway–mediated tissue development,” thereby exerting a weaker effect than downstream effector genes that directly regulate growth. Other genes within the MAPK pathway may also exhibit small or non-significant effects, making it difficult for combined association signals from individual loci to surpass the statistical threshold. This study provides a reference for further exploring the mechanism of the MAPK pathway’s role in body ruler traits in sika deer.

Furthermore, the candidate genes identified through enrichment analysis include *CDH4*, *TSHR*, *SLC23A2*, and *RIMS1*, all of which are positive regulatory functions in growth and development. At present, no direct correlation between these candidate genes and the body size trait of sika deer has been reported; their known involvement in growth-related mechanisms in other species supports their potential biological relevance. Thus, these genes provide a valuable reference framework for subsequent functional validation studies aimed at elucidating the molecular mechanisms underlying body size variation in this species.

## 5. Conclusions

In this study, 774 SNP loci significantly associated with 9 body size traits in Dongfeng sika deer bucks were identified by genome-wide association analysis. Four potential candidate genes, *CDH4*, *TSHR*, *SLC23A2*, and *RIMS1*, were screened by functional enrichment analysis for their positive regulatory functions in growth and development. This study provides a reference basis for subsequent functional exploration of candidate genes.

## Figures and Tables

**Table 1:** Statistics of Body Measurements.

Trait	Maximum	Minimum	Mean	Standard Deviation	Coefficient of Variation (%)
Body Height, cm	121	92	108.50	5.87	5.41
Body Length, cm	127	91	105.67	4.98	4.71
Chest Circumference, cm	140	57	97.19	27.60	28.40
Chest Depth, cm	54	39	47.13	3.08	6.54
Head Length, cm	41	21	30.76	3.13	10.17
Frontal width, cm	15	10	12.98	1.19	9.16
Pipe Circumference, cm	19.5	9	10.70	0.81	7.57
Tail length, cm	20	6	15.42	2.02	13.10
Antler shank distance, cm	12	7	10.08	0.97	9.60

**Table 2:** SNPs loci associated with the body size trait.

Trait	Chromosome	Location, bp	Genotype	*p*-Value	Proximity Gene
Body Height	Chr29	37,719,498	A/G	7.32 × 10^−24^	*RIC1*
	Chr25	51,359,020	G/C	7.00 × 10^−24^	none
Body Length	Chr25	15,274,058	G/C	7.63 × 10^−24^	*LOC122684034*, *LOC122683960*
	Chr27	12,003,186	G/T	7.82 × 10^−24^	*RTTN*
Chest Circumference	Chr06	105,298,383	G/A	24.53 × 10^−23^	none
	Chr08	98,463,587	G/A	28.35 × 10^−23^	none
	Chr10	2,576,269	C/T	31.67 × 10^−22^	*LOC122702151*
	Chr13	6,800,873	G/A	26.68 × 10^−23^	*UBE3A*, *LOC122707019*
	Chr14	2,063,506	A/G	34.55 × 10^−23^	*LOC122708028*
	Chr20	65,299,752	A/C	22.78 × 10^−23^	*S1PR1*, *OLFM3*
	Chr24	55,874,319	G/C	42.92 × 10^−23^	*ERC2*
	Chr24	53,493,952	G/A	23.62 × 10^−23^	*TRNAW-CCA*, *LOC122683393*
	Chr24	52,547,833	G/T	22.82 × 10^−23^	*FHIT*
	Chr28	852,575	T/C	20.38 × 10^−23^	*LOC122685417*
	Chr29	716,609	T/C	24.64 × 10^−23^	*LOC122686330*
	Chr33	149,895,086	T/C	22.81 × 10^−23^	none
Chest Depth	Chr05	18,303,189	T/C	6.70 × 10^−23^	none
	Chr20	9,754,766	T/C	7.45 × 10^−23^	none
Head Length	Chr14	2,063,506	A/G	10.37 × 10^−23^	*LOC122708028*
	Chr33	149,895,086	T/C	10.84 × 10^−22^	none
	Chr24	55,874,319	G/C	12.20 × 10^−23^	*ERC2*
	Chr23	49,007,055	G/A	9.07 × 10^−22^	*BMP2*, *LOC122681619*
	Chr26	31,522,038	G/A	9.46 × 10^−23^	*LOC122684220*
	Chr28	3,389,441	G/T	7.81 × 10^−23^	*LOC122685417*
	Chr32	11,106,917	T/C	8.20 × 10^−23^	*LOC122688080*
	Chr10	2,576,269	C/T	8.97 × 10^−23^	*LOC122702151*
Frontal Width	Chr30	1,835,247	C/T	7.68 × 10^−24^	*LOC122686995*
Pipe Circumference	Chr24	41,986,106	G/C	7.78 × 10^−24^	*FOXP1*
	Chr26	22,876,437	C/T	7.80 × 10^−23^	*ARFGEF3*
Tail Length	Chr01	31,765,591	T/A	7.24 × 10^−24^	none
	Chr11	80,720,780	G/A	7.34 × 10^−24^	SLC8A1
Shank Distance	Chr03	70,147,010	A/G	7.89 × 10^−24^	none
	Chr20	56,254,868	A/G	7.83 × 10^−24^	*LRIF1*, *LOC122676741*
	Chr23	49,421,440	A/G	7.72 × 10^−24^	*TRNAW-CCA*, *LOC122681873*

**Table 3:** GO-enriched pathways related to body size trait in Sika Deer.

GO ID	Molecular Function	*p*-Value	Gene Count	Gene	Form
GO:0034330	cell junction organization	0.0001	10	*CHRNAL*/*CDH4*/*EPHA3*/*NRXN3*/*GABRA5*/*RIMA1*/*ADGRB3d*/*ERC2*/*CDH18*/*CDH26*	BP
GO:0050890	cognition	0.0006	7	*MME*/*NRXN3*/*SORCS3*/*GPR155*/*LINS1*/*GABRA5*/*ADGRB3*	BP
GO:0016239	positive regulation of macroautophagy	0.0058	3	*SPTLC2*/*TRIM13*/*SNX7*	BP
GO:0018108	peptidyl-tyrosine phosphorylation	0.0061	3	*AGTRLA*/*EPHA3*/*LRRK1*	BP
GO:0048639	positive regulation of developmental growth	0.0072	4	*CDH4*/*TSHR*/*SLC23A2*/*RIMS1*	BP
GO:0016342	catenin complex	0.0002	3	*CDH4*/*CDH18*/*CDH26*	CC
GO:0099634	postsynaptic specialization membrane	0.0008	5	*CHRNAL*/*SORCS3*/*GLRA3*/*GABRA5*/*ADGRB3*	CC
GO:0030695	GTPase regulator activity	0.0001	8	*GDI2*/*ARHGEF3*/*ARHGAP18*/*DOCK2*/*GOPC*/*ADGRB3*/*DENND6A*/*ARFGEF3*	MF
GO:0016758	hexosyltransferase activity	0.0046	4	*GBE1*/*AGL*/*POMT2*/*GALNTL6*	MF
GO:0008238	exopeptidase activity	0.0050	3	*MME*/*CTSZ*/*SCRN3*	MF

**Table 4:** KEGG enrichment pathway analysis results.

Pathway	Database	Input Number	Background Number	*p*-Value	Corrected *p*-Value	Gene
Starch and sucrose metabolism	KEGG PATHWAY	1	33	1.64	1.01	*GBE1*
Propanoate metabolism	KEGG PATHWAY	1	36	1.61	1.01	*HIBCH*
Neuroactive ligand-receptor interaction	KEGG PATHWAY	2	372	1.59	1.01	*GLRA3*, *CHRNA1*
beta-Alanine metabolism	KEGG PATHWAY	1	38	1.59	1.01	*HIBCH*
Valine, leucine and isoleucine degradation	KEGG PATHWAY	1	52	1.45	0.97	*HIBCH*
Non-small cell lung cancer	KEGG PATHWAY	1	66	1.35	0.96	*FHIT*
Fc gamma R-mediated phagocytosis	KEGG PATHWAY	1	94	1.21	0.94	*DOCK2*
Small cell lung cancer	KEGG PATHWAY	1	100	1.18	0.94	*FHIT*
Metabolic pathways	KEGG PATHWAY	3	1540	1.08	0.94	*HIBCH*, *GBE1*, *FHIT*
Carbon metabolism	KEGG PATHWAY	1	127	1.08	0.94	*HIBCH*
Purine metabolism	KEGG PATHWAY	1	141	1.04	0.94	*FHIT*
Yersinia infection	KEGG PATHWAY	1	141	1.04	0.94	*WIPF1*
Chemokine signaling pathway	KEGG PATHWAY	1	188	0.92	0.86	*DOCK2*
Endocytosis	KEGG PATHWAY	1	269	0.77	0.74	*WIPF1*
MAPK signaling pathway	KEGG PATHWAY	1	305	0.73	0.73	*MAP3K20*

## Data Availability

The original contributions presented in the study are included in the article/Supplementary Materials; further inquiries can be directed to the corresponding author.

## Supplementary Materials

The following supporting information can be downloaded at: https://www.mdpi.com/article/10.3390/biology15030227/s1, Table S1: SNP information based on MLM analysis, associated with BL; Table S2: Loci of SNPs associated with some body size traits.
